# The Gut-Muscle Axis in Older Subjects with Low Muscle Mass and Performance: A Proof of Concept Study Exploring Fecal Microbiota Composition and Function with Shotgun Metagenomics Sequencing

**DOI:** 10.3390/ijms21238946

**Published:** 2020-11-25

**Authors:** Andrea Ticinesi, Leonardo Mancabelli, Sara Tagliaferri, Antonio Nouvenne, Christian Milani, Daniele Del Rio, Fulvio Lauretani, Marcello Giuseppe Maggio, Marco Ventura, Tiziana Meschi

**Affiliations:** 1 Geriatric-Rehabilitation Department, Azienda Ospedaliero-Universitaria di Parma, Via Antonio Gramsci 14, 43126 Parma, Italy; anouvenne@ao.pr.it (A.N.); fulvio.lauretani@unipr.it (F.L.); marcellogiuseppe.maggio@unipr.it (M.G.M.); tiziana.meschi@unipr.it (T.M.); 2 Microbiome Research Hub, University of Parma, Parco Area delle Scienze 11/A, 43124 Parma, Italy; leonardo.mancabelli@unipr.it (L.M.); christian.milani@unipr.it (C.M.); marco.ventura@unipr.it (M.V.); 3 Department of Chemistry, Life Sciences and Environmental Sustainability, Parco Area delle Scienze 11/A, 43124 Parma, Italy; 4 Department of Medicine and Surgery, University of Parma, Via Antonio Gramsci 14, 43126 Parma, Italy; sara.tagliaferri@unipr.it; 5 Department of Medical Veterinary Sciences, University of Parma, Strada del Taglio 10, 43126 Parma, Italy; daniele.delrio@unipr.it

**Keywords:** gut-muscle axis, frailty, gut microbiota, physical function, geriatrics

## Abstract

The gut microbiota could influence the pathophysiology of age-related sarcopenia through multiple mechanisms implying modulation of chronic inflammation and anabolic resistance. The aim of this study was to compare the fecal microbiota composition and functionality, assessed by shotgun metagenomics sequencing, between two groups of elderly outpatients, differing only for the presence of primary sarcopenia. Five sarcopenic elderly subjects and twelve non-sarcopenic controls, classified according to lower limb function and bioimpedance-derived skeletal muscle index, provided a stool sample, which was analyzed with shotgun metagenomics approaches, to determine the overall microbiota composition, the representation of bacteria at the species level, and the prediction of bacterial genes involved in functional metabolic pathways. Sarcopenic subjects displayed different fecal microbiota compositions at the species level, with significant depletion of two species known for their metabolic capacity of producing short-chain fatty acids (SCFAs), *Faecalibacterium prausnitzii* and *Roseburia inulinivorans*, and of *Alistipes shahii*. Additionally, their fecal metagenome had different representation of genes belonging to 108 metabolic pathways, namely, depletion of genes involved in SCFA synthesis, carotenoid and isoflavone biotransformation, and amino acid interconversion. These results support the hypothesis of an association between microbiota and sarcopenia, indicating novel possible mediators, whose clinical relevance should be investigated in future studies.

## 1. Introduction

Sarcopenia, defined as the loss of skeletal muscle mass and function typical of aging occurring in the absence of any identifiable cause, represents a relevant health condition increasing the risk of disability and mortality [1]. The pathophysiology of this condition is not fully understood, but the presence of age-related chronic inflammation and anabolic resistance seem to play a major role, especially when combined with behavioral risk factors for muscle wasting such as inactivity and malnutrition [2,3].

Sarcopenia is frequently overlapped with frailty, a geriatric syndrome characterized by reduced homeostatic reserves increasing the risk of adverse outcomes [4]. Frailty can be either diagnosed as a clinical phenotype characterized by fatigue, weight loss, sedentary behavior, and reduction of physical function, or as the accumulation of deficits in physiological functions [5]. Inflammation and anabolic resistance play an important pathophysiological role also in frailty, so sarcopenia can represent the biological substrate of the clinical syndrome of frailty [6]. In this perspective, both conditions have been recently merged into a new entity, called physical frailty and sarcopenia (PF&S) [7].

The gut microbiota is being increasingly recognized as a modulator of inflammatory response and anabolic balance [8,9,10]. Gut microbiota dysbiosis, implying disruption of the equilibrium of microbial communities with overrepresentation of opportunistic pathogens and depletion of symbionts and commensals, could thus represent a trigger for inflammation and anabolic resistance [8,9,10]. This condition is particularly frequent in older individuals, where malnutrition, inactivity, chronic diseases, and polypharmacy can contribute to enhance dysbiosis [11,12,13,14]. Conversely, individuals with successful aging, such as centenarians, show specific signatures of health in their intestinal microbiota, with elevated representation of bacteria associated with anti-inflammatory properties, such as Bifidobacteria [15,16,17].

Several researchers have hypothesized that the intestinal microbiota could either represent a biomarker of pathophysiological mechanisms contributing to the development of PF&S, or an active modulator of skeletal muscle function during aging [8,9,10,18,19]. In this perspective, modulation of the microbiota through dietary intervention or administration of probiotics or prebiotics may represent an interesting target to counterbalance age-related loss of muscle mass and function [18].

The existence of a gut-muscle axis modulating skeletal muscle mass and function has been recently demonstrated in animal models [20,21,22]. The transplantation of fecal microbiota from fit older humans to mice resulted in improved muscle function, unlike the transplantation of fecal microbiota from older humans with impaired muscle function [22].

To date, few studies have explored the gut-muscle axis in human beings. However, the abundance of important components of human fecal microbiota, such as *Enterobacteriaceae*, *Bacteroides*, and *Prevotella*, has been correlated with measures of muscle fitness, such as gait speed [10]. Moreover, a large population-based study has demonstrated that gut microbiota dysbiosis may be associated with frailty, measured in accordance with the deficit accumulation model [23].

Recently, Picca et al. have shown that the fecal microbiota from older individuals with PF&S is characterized by increased representation of *Bifidobacteriaceae*, *Dialister*, *Pyramidobacter*, and *Eggerthella* and depletion of *Slackia* and *Eubacterium* in comparison with non-frail matched controls, and that these alterations are associated with increased levels of pro-inflammatory cytokines and a distinct serum metabolic profile [24].

These findings support the gut-muscle axis hypothesis, but still leave room for uncertainty. First, the metabolic functions of the gut microbiota of older subjects with PF&S have not been investigated yet. Data from mouse models also suggest that sarcopenia may be associated with reduced capacity of biotransformation of nutrients such as amino acids and folic acid [20]. The interaction of microbiota with diet, with particular regard to protein intake, also needs clarification for assessing the true impact of the microbiota on skeletal muscle physiology [25,26,27].

The aim of the present study was to assess the feasibility of comparing the fecal microbiota composition and functionality with a shotgun metagenomics sequencing approach between two small groups of older community-dwellers, differing only in their muscle mass and lower limb performance.

## 2. Results

### 2.1. Clinical and Nutritional Characteristics of Participants

Seventeen patients (three males and 14 females) provided stool samples for microbiome analysis and completed the clinical procedures of the study. Five were classified as sarcopenic and physically frail (1 male, 4 females) and twelve as non-sarcopenic and fit (2 males, 10 females), according to the criteria detailed in the Methods section. The overall demographical and clinical characteristics of participants are summarized for descriptive purposes in Table 1. Namely, sarcopenic and non-sarcopenic subjects were similar for age, weight, Body Mass Index (BMI), and the Bristol Stool Chart Score. They instead differed in Skeletal Muscle Index (SMI), Skeletal Muscle Mass (SMM), and Short-Physical Performance Battery (SPPB) score, as expected according to the inclusion criteria.

The average nutritional intakes of macronutrients and micronutrients, estimated through the EPIC food-frequency questionnaire (FFQ), were similar between sarcopenic and non-sarcopenic participants, as detailed in Table 2. Thus, the two groups of participants differed only in skeletal muscle mass and performance of lower limbs.

### 2.2. Composition of the Fecal Microbiota

Shallow-shotgun metagenomics analysis of the fecal samples produced an average number of 44,827 ± 21,635 reads per sample. After quality filtering and *Homo sapiens* filtering, the average number of reads that were taxonomically classified was 18,173 ± 9122 per sample. Taxonomical classification of reads at the species level was completed only for reads with an abundance >0.1% in at least one sample.

**Figure 1:**
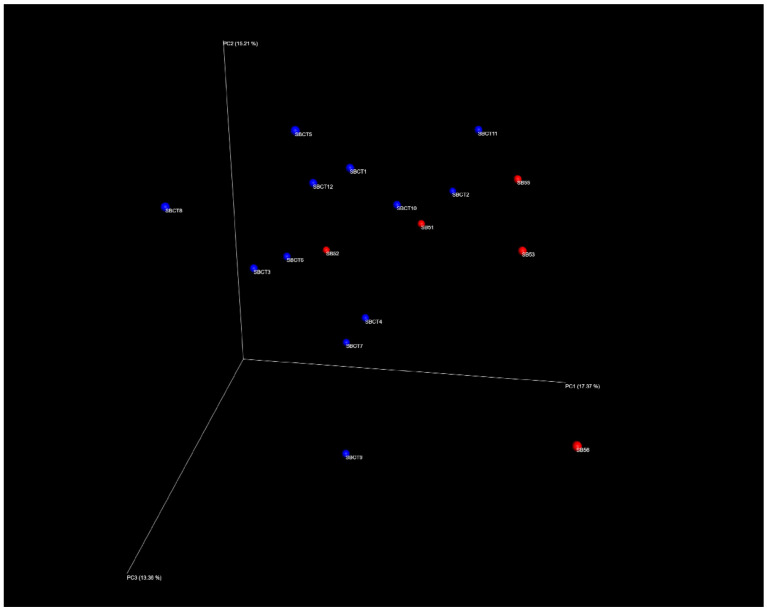
Principal Coordinate Analysis (PCoA) plot showing the differences in the microbiota composition of each fecal sample (inter-individual variability) on three-dimension axes. Each dot corresponds to the unique composition of a single fecal sample. The distance between dots represents the degree of variability of the microbiota composition among samples. Samples from sarcopenic subjects (n = 5) are indicated in red and samples from control subjects (n = 12) are indicated in blue. No statistically significant clusters could be identified through PERMANOVA and ANOSIM tests.

The index of species richness, i.e., biodiversity, obtained considering only these reads was on average similar between sarcopenic and non-sarcopenic participants (78 ± 28 vs. 81 ± 18, *p* = 0.8). The overall composition of the microbiota was also compared between sarcopenic and non-sarcopenic participants using Principal Coordinate Analysis (PCoA) (Figure 1). The elevated distance of each dot in the PCoA plot suggested the presence of high inter-individual variability among all study participants, as expected for older individuals. Although visual inspection of the PCoA plot may suggest the presence of two different clusters of microbiota composition, corresponding to sarcopenic and non-sarcopenic participants (Figure 1), the statistical analysis conducted with PERMANOVA and the ANOSIM test retrieved no significant differences (*p* = 0.36).

**Figure 2:**
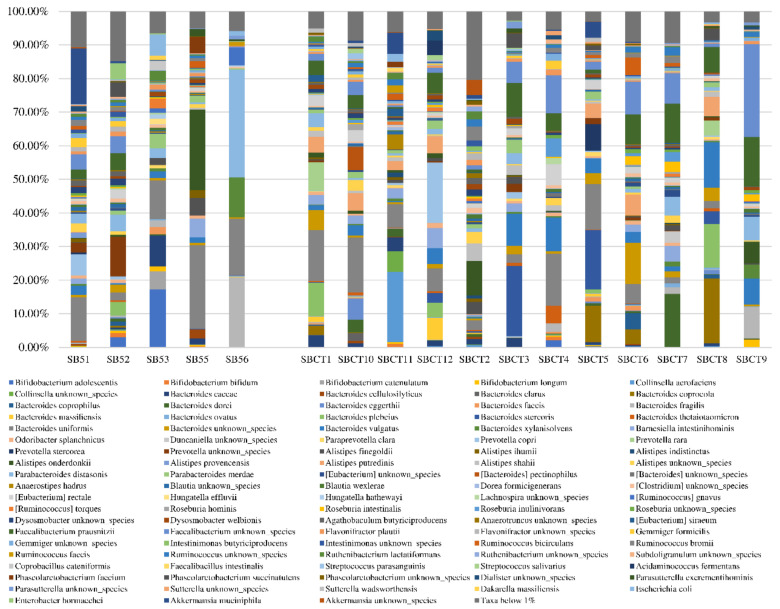
Graphical representation of the fecal microbiota composition, assessed by shotgun metagenomics sequencing, of five older subjects with primary sarcopenia (left) and 12 non-sarcopenic controls (right). Only species with a relative abundance >1% in at least one sample are included in this graphical representation.

The microbiota composition, in terms of the relative abundance of the most represented species, is depicted for each sample in Figure 2. The relative abundance of 251 different bacterial species was compared between sarcopenic and non-sarcopenic participants with the Mann–Whitney test. The full list of species is provided in Supplementary File S1. The comparison retrieved significant results for three bacterial species, *Faecalibacterium prausnitzii, Roseburia inulinivorans*, and *Alistipes shahii*, that were significantly more represented in the fecal samples from non-sarcopenic than sarcopenic participants (Table 3). Namely, the relative representation of *F. prausnitzii* in non-sarcopenic subjects was five times higher than in subjects with primary sarcopenia (median 5.56%, IQR 1.79–9.87%, vs. 0.15%, IQR 0.07–3.93%, *p* = 0.19).

### 2.3. Function of the Fecal Microbiota

Shotgun metagenomics sequencing allowed us to verify the presence of 3186 different MetaCyc pathways in the bacterial metagenome of each fecal sample (full list provided in Supplementary File S2). An average representation of at least 0.01% of the whole metagenome was found for 1579 MetaCyc pathways (full list provided in Supplementary File S2). A comparison of the average representation of each of these 1579 pathways between sarcopenic and non-sarcopenic participants retrieved 108 significant results with a *p*-value < 0.05 (Table 4, full list provided in Supplementary File S2).

In comparison with controls, the fecal metagenome of sarcopenic participants was significantly depleted of genes involved in amino acid metabolism (glutamine and isoleucine degradation, methionine, lysine, and threonine biosynthesis), alpha carotene biosynthesis, flavin biosynthesis, and pyruvate fermentation to acetate (Table 4). Conversely, the fecal metagenome of sarcopenic participants was enriched with genes involved in glycolysis and glyoxylate bypass (Table 4).

## 3. Discussion

In a small group of older subjects with reduced muscle mass and lower limb performance, we were able to detect statistically significant differences in the composition and functionality of the fecal microbiota, in comparison with a group of controls with similar age, clinical characteristics, and dietary habits. The statistically significant differences did not involve the overall biodiversity of intestinal microbiota, but mainly regarded the abundance of a reduced number of bacterial species and the expression of a list of genes involved in several pathways of bacterial metabolism.

These results should be carefully considered in the light of the reduced sample size of the study, with only five sarcopenic participants mostly of female gender. From one perspective, these characteristics of the study design represent an important limitation, because they probably prevented adequate assessment of differences in microbiota biodiversity and inter-individual variability related to the presence of physical frailty and sarcopenia. Such assessment would have required a larger sample size and more homogeneity in the representation of cases and controls. However, the presence of some statistically significant differences in the context of such a reduced sample size supports the plausibility of the existence of a gut-muscle axis in the pathophysiology of physical frailty and sarcopenia, matches the findings obtained in mouse models, and represents a basis for the design of future studies in this field.

The fecal microbiota could influence the skeletal muscle metabolism at multiple levels: modulating the intestinal absorption of nutrients involved in protein synthesis, regulating anabolic balance and insulin resistance through different microbial mediators, and promoting the onset of chronic systemic inflammation [9,14]. Although in the present investigation the differences in microbiota composition between sarcopenic participants and controls were not significant in terms of beta-diversity, we could identify several meaningful trends worthy of investigation in future studies with larger sample sizes. Namely, the circumstance that the abundance of *F. prausnitzii* was up to five times lower in the fecal samples of subjects with sarcopenia suggests that the depletion of this bacterial species represents a distinctive feature of the gut microbiota in patients with sarcopenia.

*F. prausnitzii* is widely known as one of the key health-promoting components of the gut microbiota, since its abundance is associated with improved insulin sensitivity, anabolic balance, and alleviation of local intestinal and systemic inflammation [28,29,30]. Alterations of the intestinal abundance of *F. prausnitzii* correlate with markers of inflammation in both humans with chronic inflammatory bowel diseases and mouse models [31,32,33,34]. These actions are largely dependent on the production of short-chain fatty acids (SCFAs), namely, butyrate, by *Faecalibacterium*. SCFAs can be absorbed into systemic circulation and modulate a wide range of physiological systems, including skeletal muscle protein synthesis [35]. Recent evidence from mouse models suggests that gut microbiota dysbiosis, with reduced representation of bacterial species involved in SCFA synthesis, is also associated with reduced expression of free fatty acid receptors in the intestinal mucosa enteroendocrine cells, with detrimental consequences for the skeletal muscle cell glucose metabolism (i.e., glycogen depletion, anabolic resistance) [36].

Interestingly, the administration of SCFAs, and particularly butyrate, to mouse models of aging resulted in prevention of age-related muscle mass loss or even in promotion of muscle synthesis [37,38]. Moreover, depletion of *F. prausnitzii* in the fecal microbiota of older subjects suffering from physical frailty and sarcopenia was also one of the findings of the study by Picca et al., who compared the microbiota composition between subjects with and without physical frailty and sarcopenia by using a 16S rRNA microbial profiling approach [24]. Finally, a recent study conducted on patients with normal-weight obesity showed that the abundance of *F. prausnitzii* was inversely correlated with skeletal muscle mass [39].

*R. inulinivorans* and *A. shahii*, the other two bacterial species found as significantly depleted in the fecal microbiota of sarcopenic subjects, could also play a protective role in the pathophysiology of sarcopenia. Bacteria belonging to the genus *Roseburia*, including *R. inulinivorans*, are well known SCFA producers [40]. *R. inulinivorans* is particularly effective in transforming dietary sugars into propionate [41], a compound whose physiological functions are similar to those of butyrate, although perhaps less studied [35]. In patients suffering from active forms of inflammatory bowel disease, depletion of *R. inulinivorans* and *F. prausnitzii* often co-occurs, implying a possible role of both species as modulators of inflammation [42]. *A. shahii* has instead a less defined metabolic role in the gut microbiota, although depletion of bacteria belonging to the *Alistipes* genus is generally considered an important marker of gut microbiota dysbiosis [43], as observed by our research group in elderly hospitalized patients developing *Clostridium difficile* enterocolitis [44]. Interestingly, depletion of *A. shahii* in the fecal microbiota has been recently associated with adverse outcomes and death in a group of Chinese centenarians [45].

The impact of these components of the intestinal microbiota on the skeletal muscle health remains however to be precisely determined. Dietary habits could play an important role in this relationship, since the capacity of *Roseburia* and *Faecalibacterium* to produce SCFAs is largely dependent on the intake of complex sugars and fiber, such as inulin [46]. Moreover, the capacity of *F. prausnitzii* to produce butyrate also depends on cross-feeding interactions with other members of the intestinal microbial communities, and particularly with Bifidobacteria [47,48]. In fact, the butyrate synthesis by F. prausnitzii is possible only in presence of an adequate amount of *Bifidobacterium adolescentis* and *Bifidobacterium longum* [47,48], which were present in small relative abundances in both the sarcopenic and non-sarcopenic participants of our investigation. However, the results of the comparison of functional pathways expressed by the fecal microbiota (Table 4) suggest that the capacity of producing SCFAs, namely, acetate, may really be decreased in sarcopenic subjects.

Another key result of the shotgun metagenomics sequencing performed in our study is represented by the differences in microbiome functionality between sarcopenic and control subjects. The genes corresponding to several metabolic pathways were less represented in the sarcopenic fecal metagenome than in controls, and this aspect may be worthy of further investigation in larger prospective studies.

For example, the reduced capacity of the sarcopenic fecal metagenome to produce alpha-carotene may imply lower antioxidant capacity of the host and reduced stimulation for skeletal muscle synthesis [49]. Dietary intake of carotenoids is positively related to skeletal muscle mass in older individuals, and a role of the gut microbiota in this relationship is probable [50]. Furthermore, population-based studies have shown that reduced serum levels of carotenoids can be considered a biomarker of sarcopenia [51], and carotenoids have been proposed as a protective agent against sarcopenia since the early 2000s [52].

The fecal microbiome of sarcopenic subjects was also less enriched with functionalities involved in amino acid metabolism, as compared with controls. This circumstance suggests that the gut microbiota may be involved in regulating amino acid absorption by the gut mucosa, and thus the amount of substrates available for protein synthesis in the skeletal muscle cells. These findings are coherent with the results of a study conducted in mouse models of sarcopenia by Siddhart et al. [20], but their precise implications for human skeletal muscle metabolism remain to be determined. However, reduced bioavailability of branched-chain amino acids, such as isoleucine, and beta-alanine in the intestinal microenvironment, suggested by the findings shown in Table 4, could have relevance for the sarcopenia pathophysiology. In older individuals, low availability of branched-chain amino acids is associated with reduced muscle mass and function [53], while these parameters are improved by dietary supplementation [54]. Exogenous beta-alanine supplementation can improve endurance capacity and functional performance in elderly subjects [55], thus it seems reasonable to hypothesize that a reduced biosynthetic capacity of this compound by the gut metagenome has negative consequences for muscle function.

Finally, the microbiome of sarcopenic subjects was significantly depleted of some functionalities involved in isoflavone metabolism, such as daidzein interconversion (Table 4). These molecules could also represent important elements of the gut-muscle axis in sarcopenia, since they are able to down-regulate ubiquitin-specific protease in skeletal muscle cells [56]. For example, evidence from mouse models suggest that daidzein is able to increase skeletal muscle mass by reducing proteolysis in muscular cells [56].

Overall, the results of our study, to our knowledge the first one conducted in sarcopenic older subjects with a shotgun metagenomics sequencing approach, provide important preliminary data that could represent the bases for future, larger studies exploring more comprehensively and in depth the relationships between gut microbiota and the presence of physical frailty and sarcopenia. These results should, however, be interpreted with caution due to some relevant study limitations. The most obvious one, as discussed above, is the reduced sample size, that prevented us from obtaining statistical significant differences in comparisons for several clinical, nutritional, and microbiological variables and to test correlations. The cross-sectional design of the study also prevented us from drawing any conclusion on causality between gut microbiota alterations and the presence of reduced muscle mass and performance. The assessment of muscle mass of participants, though coherent with the current European guidelines [1], was not made with gold-standard or first-choice techniques (magnetic resonance or dual-energy X-ray absorptiometry, respectively). However, the use of bioimpedance analysis (BIA) seems reasonable in the outpatient context, where confounding factors such as acute illness and hydro-electrolyte imbalances are absent [57,58]. Finally, participants were mainly of female gender, and this circumstance prevented us from identifying possible gender-related differences in the association between the microbiome and muscle mass and function.

## 4. Materials and Methods

### 4.1. Study Design, Participants, and Setting

Seventeen subjects attending the outpatient Frailty Clinic of Geriatric-Rehabilitation Department, Parma University-Hospital, for comprehensive geriatric assessment were enrolled in this proof-of-concept study. The proof-of-concept design was chosen because the intent was to generate preliminary data from a small set of patients enrolled in a highly controlled setting, in order to provide useful information for the design of future studies testing the gut-muscle axis hypothesis in larger populations.

Included were subjects aged ≥70 years old living in the community, not recently hospitalized. Exclusion criteria were residence in nursing homes, disability with severe impairment in Activities of Daily Living (ADL) and Instrumental Activities of Daily Living (IADL), presence of medical conditions causing secondary sarcopenia (such as stroke, severe malnutrition, osteoarthritis or other rheumatic diseases), neurological diseases including cognitive impairment or dementia, cancer, chronic constipation, gastrointestinal diseases with known association with gut microbiota dysbiosis (inflammatory bowel disease, coeliac disease, cirrhosis), chronic treatment with statins or other drugs with known myotoxicity, such as steroids, presence of restrictive dietary regimens, and administration of antibiotics in the month before assessment. Thus, all patients with a possible secondary cause of sarcopenia were excluded from the study.

Participants were classified as physically frail and sarcopenic when fulfilling the following two criteria: reduced lower limb function, assessed by SPPB score between 3/12 and 9/12, and reduced muscle mass, determined by bioimpedance analysis with SMI/height^2^. Conversely, participants were classified as fit (non-sarcopenic) when they had normal lower limb function (SPPB score between 10/12 and 12/12), and normal muscle mass. SMI was calculated using Janssen et al.’s equation [59,60] and considering the cut-offs recommended by the first European Consensus on Sarcopenia (<8.87 kg/m^2^ in men and <6.42 kg/m^2^ in females) for defining reduced muscle mass [61]. Subjects fulfilling only one of the two criteria for sarcopenia (i.e., low SPPB score with normal muscle mass, or normal SPPB score with reduced muscle mass) were not included in the final analysis.

These criteria allowed us to compare two groups of subjects with truly different muscle mass and function, but with similar clinical characteristics.

The study protocol was approved by the local Ethics Committee (Comitato Etico per Parma), under the ID 37136 (approval date 10/18/2017) and authorized by the Parma University-Hospital Direction. Written informed consent for participation was obtained from all participants.

### 4.2. Study Procedures

Each participant received a complete clinical examination and comprehensive geriatric assessment. Namely, the motoric evaluation included the measurement of grip strength of the dominant side by a hand-held hydraulic dynamometer, measurement of gait speed on a 4-m linear path, chair-stand test, and balance test in the tandem and semitandem positions. The latter three tests were combined for calculating the SPPB score, with each test ranking from 0 (=incapacity to perform the test in due time) to 4 (=normal performance) and the total SPPB score resulting from the sum of each of the three sub-scores [62]. Cognitive function was assessed with the Mini Mental State Examination (MMSE) test.

The evaluation of the dietary habits of participants was also completed with a semi-quantitative FFQ validated for the Italian population in the context of the European Prospective Investigation on Cancer study (EPIC questionnaire) [63], that has already been used for investigating food intake in the geriatric setting [64] and in gut microbiome studies [65]. This questionnaire provides a comprehensive overview of dietary habits of the last year, allowing a reliable determination of the intake of macronutrients and micronutrients [66]. It was administered by a trained nutritionist following a consolidated methodology, allowing us to make a reliable interpretation of participants’ responses avoiding possible incoherence. A detailed description of the structure of the FFQ was described in the validation study [66]. The decoding of the questionnaire was performed automatically through the service provided by the Italian EPIC center of reference, located at the Italian National Cancer Institute. Basically, nutrient intake was calculated according to food tables which were compiled and are continuously updated for the purpose of nutrition surveys by the national Council for Agricultural Research and Analysis of Agricultural Economics (https://www.alimentinutrizione.it).

Each patient also underwent BIA using the BIA 101 device (Akern Srl, Florence, Italy). The test was performed with participants lying supine with their limbs slightly away from their body, after bladder voiding. Active electrodes (BIATRODES^®^ Akern 161 Srl; Florence, Italy) were placed on the right side on conventional metacarpal and metatarsal lines.

At each location, the BIA 101 device, operating at a weak alternating electrical current of 500 mA and a single frequency of 50 kHz, was employed to measure the voltage drop across body tissues. Skeletal muscle mass (SMM) was calculated using the BIA equation by Janssen and colleagues [60], and its derivative end-point, SMI, was obtained by dividing SMM by height squared (SMI = kg/m^2^). Height was measured with the help of a stadiometer.

Finally, on the same day of clinical and nutritional investigations, each patient handed over a 2-g fecal sample for gut microbiota analyses. The samples were self-collected in the morning by spontaneous evacuation and stored at room temperature in stool nucleic acid collection and preservation tubes (Norgen Biotek, Thorold, Niagara, Ontario, Canada) containing 2 mL of preservative and inactivating solution. Samples were then delivered by participants to the research center within 24 h and then refrigerated at −22 °C. The Bristol Stool Chart classification of the provided fecal samples was collected for each participant as additional information, through visual inspection and focused interview.

### 4.3. Microbiota Analyses

Stool samples were analyzed at the Microbiome Research Hub facilities of the University of Parma using a shallow-shotgun metagenomics approach. Bacterial DNA was extracted from fecal samples using the QIAamp Fast DNA Stool Mini kit following the manufacturer’s instructions (Qiagen Ltd., Strasse, Germany) and quantified using fluorometric Qubit quantification system (Life Technologies, Thermo Fisher Scientific, Waltham, Massachusetts, USA). A DNA library was prepared using the Nextera XT DNA sample preparation kit (Illumina, San Diego, California, USA) according to the manufacturer’s instructions. In detail, one ng input DNA from each sample was used for library preparation. The isolated DNA underwent enzymatically fragmentation, adapter ligation, and purification involving magnetic beads. Then, samples were quantified using a fluorometric Qubit quantification system (Life Technologies, Thermo Fisher Scientific, Waltham, Massachusetts, USA) loaded on a 2200 Tape Station Instrument (Agilent Technologies, Santa Clara, California, USA) and normalized to 4 nM. Sequencing was performed on a MiSeq instrument (Illumina, San Diego, California, USA), according to the manufacturer’s instructions, using the 2 × 250 MiSeq Reagent Kit v3 (600-cycle), and spike-in of 1% PhiX control library.

Taxonomic profiling of sequenced reads was performed with the METAnnotatorX bioinformatics platform (Computational Microbiology Unit, University of Parma, Parma, Italy) [67]. In detail, the raw data in fastq format were submitted to quality filtering with removal of reads with an average quality <25. Subsequently, host DNA was removed by reads mapping to the human genome. Retained sequences were used as input to perform a MegaBLAST [68] local alignment of reads to pre-processed database including available genomes of eukaryotes (Fungi and Protists), bacteria, archaea, and viruses. Reads showing a nucleotide identity >94% to the genomes included in the database were classified at the species level, while if a lower percentage identity was detected, they were classified at the genus level as undefined species. These cut-offs are those generally employed for the ANI taxonomic assignment of genomes [69,70].

Functional profiling of sequenced reads was performed with the METAnnotatorX bioinformatics platform [68]. Functional classification analyses of reads were performed to reveal metabolic pathways based on the MetaCyc classification [71].

Similarities between samples (beta-diversity) were calculated by Bray–Curtis dissimilarity based on species abundance. The range of similarities was calculated between values 0 and 1. PCoA representations of beta-diversity were performed using QIIME2 [72,73]. In the PCoA, each dot represented a sample distributed in tridimensional space according to its own bacterial composition.

### 4.4. Statistical Analyses

Data were expressed as the median and interquartile range (IQR) or percentages, as appropriate. The representation of each bacterial species and group of bacterial genes involved in similar cellular or metabolic functions was expressed as relative abundance of the overall microbiota composition and function for each sample.

Demographical, clinical, and nutritional data were compared between sarcopenic participants and controls with the Mann–Whitney test. The index of biodiversity, expressed as the number of species detected by shallow-shotgun metagenomics approach in each stool sample, and the relative abundances of species with representation >0.01% in at least one sample were also compared between cases and controls with the Mann–Whitney test. The overall composition of the fecal microbiota, in terms of biodiversity, was compared between cases and controls using PERMANOVA and ANOSIM tests. Finally, the representation of MetaCyc pathways in the fecal microbiota was compared between cases and controls using a *t* test. *p* values were considered significant when <0.05. Analyses were performed with the SPSS and R statistical packages.

## 5. Conclusions

The fecal microbiome of a small group of older patients with reduced muscle mass and lower limb performance had a different composition and functionality in comparison with the fecal microbiome of fit controls of similar age. Biosynthesis of SCFAs, carotenoids, and isoflavones and the interconversion of amino acids were the main functionalities of the gut microbiome exhibiting a significant depletion in sarcopenic patients. Larger studies should investigate these pathophysiological mechanisms in the future to assess the true relevance of the gut-muscle axis for the development of age-related sarcopenia.

## Figures and Tables

**Table 1:** Comparison of the main clinical characteristics between sarcopenic and non-sarcopenic participants.

Variable	Sarcopenic Subjects (n = 5)	Non-Sarcopenic Controls (n = 12)	*p* *
Age, years	77 (75.5–86)	71.5 (70–75)	0.08
SPPB, points	6 (3–8)	11 (10–12)	**<0.001**
SMM, Kg	14.6 (13.7–15.8)	18.2 (17.1–23.5)	**<0.001**
SMI, Kg/m^2^	6.40 (6.33–6.47)	7.24 (7.04–9.44)	**<0.001**
Weight, Kg	59.5 (45.1–70.4)	66.1 (61.3–78.5)	0.165
BMI, Kg/m^2^	24.3 (20.9–26.7)	27.4 (24.5–29.1)	0.075
Bristol Stool Scale, points	4 (1.5–5.5)	3 (2.5–5)	0.970

* Calculated with the Mann–Whitney test. Data expressed as median and interquartile range. Significant *p* values (<0.05) are indicated in bold. SPPB = Short Physical Performance Battery; SMM = Skeletal Muscle Mass; SMI = Skeletal Muscle Index; BMI = Body Mass Index.

**Table 2:** Comparison of the main daily nutritional intakes, calculated through the EPIC food-frequency questionnaire, between sarcopenic and non-sarcopenic participants.

Variable/Nutrient	Sarcopenic Subjects (n = 5)	Non-Sarcopenic Controls (n = 12)	*p* *
Total proteins, g	55.5 (46.1–92.9)	76.6 (56.7–93.5)	0.69
Animal proteins, g	30.4 (27.7–58.3)	42.6 (22.2–64.9)	0.99
Vegetal proteins, g	27.8 (17.1–34.5)	25.3 (22.3–32.3)	0.89
Total lipids, g	79.8 (46.2–105.4)	89.2 (75.4–96.5)	0.70
Animal lipids, g	30.7 (27.6–51.6)	32.1 (21.5–48.6)	0.60
Vegetal lipids, g	49.2 (18.8–53.9)	55.2 (44.4–67.5)	0.29
Total saturated lipids, g	24.7 (17.5–35.0)	25.2 (18.6–30.5)	0.99
Total polyunsaturated lipids, g	10.4 (5.8–13.8)	10.8 (8.7–13.6)	0.79
Cholesterol, mg	216 (196–305)	228 (168–323)	0.99
Sugars, g	238 (169–290)	229 (184–271)	0.89
Fibers, g	22.2 (14.1–30.0)	21.5 (18.4–23.5)	0.89
Energy, Kcal	1873 (1236–2460)	1971 (1714–2257)	0.69
Iron, mg	8.77 (5.61–14.00)	9.90 (8.89–12.64)	0.43
Calcium, mg	548 (496–1064)	891 (616–992)	0.44
Sodium, mg	1915 (1547–2959)	1806 (1596–2161)	0.90
Potassium, mg	2587 (1637–3792)	2989 (2818–3748)	0.44
Zinc, mg	6.6 (5.8–10.4)	8.9 (6.6–11.7)	0.40
Tiamin, mg	0.58 (0.40–0.96)	0.92 (0.85–1.40)	0.43
Riboflavin, mg	0.63 (0.52–1.02)	0.92 (0.85–1.40)	0.08
Niacin, mg	12.09 (8.47–20.53)	18.17 (13.41–21.65)	0.43
Vitamin C, mg	140 (92–177)	159 (111–177)	0.44
Vitamin B_6_, mg	1.29 (0.70–1.91)	1.64 (1.34–2.29)	0.24
Folic acid, μg	231 (147–343)	276 (239–348)	0.51
Beta-carotene, μg	2729 (808–3403)	3652 (2868–5147)	0.11
Vitamin E, μg	13.1 (6.2–16.5)	16.0 (13.6–19.9)	0.24
Vitamin D, mg	1.39 (1.27–3.32)	2.41 (1.29–4.21)	0.60

* Calculated with the Mann–Whitney test. Data expressed as median and interquartile range.

**Table 3:** Selection of the main bacterial species identified through shallow shotgun metagenomics sequencing in fecal samples of sarcopenic and non-sarcopenic participants and comparison of their relative abundance between the two groups. The full list of detected bacterial species is provided in Supplementary File S1.

Bacterial Species	Sarcopenic Subjects (n = 5)	Non-Sarcopenic Controls (n = 12)	*p* *
*Akkermansia muciniphila*	0.00% (0.00–8.62)	0.00% (0.00–0.09)	0.99
*Alistipes onderdonkii*	0.29% (0.00–12.35)	0.60% (0.14–1.40)	0.57
*Alistipes shahii*	0.00% (0.00–0.20)	0.88% (0.16–1.70)	**0.019**
*Bacteroides caccae*	0.44% (0.00–5.50)	1.01% (0.08–2.44)	0.87
*Bacteroides dorei*	0.16% (0.00–0.68)	0.46% (0.18–1.97)	0.23
*Bacteroides fragilis*	0.42% (0.09–11.39)	0.26% (0.04–1.59)	0.50
*Bacteroides uniformis*	13.02% (6.93–20.78)	6.33% (2.26–14.78)	0.19
*Bacteroides vulgatus*	1.67% (0.17–2.34)	3.78% (1.47–9.13)	0.08
*Barnesiella intestinihominis*	0.12% (0.00–3.61)	2.38% (0.11–2.93)	0.44
*Bifidobacterium longum*	0.42% (0.19–1.08)	0.00% (0.00–0.38)	0.13
*Escherichia coli*	0.26% (0.06–3.83)	0.00% (0.00–0.28)	0.16
*Faecalibacterium prausnitzii*	0.15% (0.07–3.93)	5.56% (1.79–9.87)	**0.019**
*Flavonifractor plautii*	0.93% (0.61–1.42)	0.52% (0.31–0.94)	0.23
*Parabacteroides distasonis*	2.94% (1.68–18.49)	1.06% (0.61–3.92)	0.32
*Parabacteroides merdae*	1.22% (0.17–3.21)	1.14% (0.17–2.16)	0.87
*Roseburia intestinalis*	0.29% (0.00–0.41)	0.32% (0.03–1.70)	0.51
*Roseburia inulinivorans*	0.00% (0.00–0.00)	0.32% (0.12–0.97)	**0.006**
*Ruminococcus bromii*	0.89% (0.00–1.50)	0.32% (0.04–1.43)	0.99
*Ruminococcus gnavus*	0.33% (0.07–3.28)	0.14% (0.00–0.25)	0.19
*Subdoligranulum* unknown species	0.17% (0.00–0.44)	0.21% (0.12–0.36)	0.64

* Calculated with the Mann–Whitney test. Data expressed as median and interquartile range of the relative abundance on the overall microbiota composition. Significant *p* values (<0.05) are indicated in bold.

**Table 4:** Selection of the main MetaCyc pathways expressed by genes of the fecal metagenome in the analyzed fecal samples and comparison of their average representation between sarcopenic and non-sarcopenic subjects.

Variable/Nutrient	Sarcopenic Subjects (n = 5)	Non-Sarcopenic Controls (n = 12)	*p* *
Alpha-carotene biosynthesis	0.13 ± 0.03	0.18 ± 0.04	**0.049**
Beta-alanine biosynthesis	0.02 ± 0.02	0.06 ± 0.03	**0.023**
Acetyl-CoA fermentation to butanoate	0.23 ± 0.06	0.32 ± 0.08	**0.036**
Daidzein conjugates interconversion	0.34 ± 0.03	0.41 ± 0.07	**0.048**
Flavin biosynthesis	0.05 ± 0.04	0.11 ± 0.04	**0.018**
Glycolysis I (from glucose-6-phosphate)	0.85 ± 0.21	0.64 ± 0.09	**0.009**
L-glutamine degradation	0.59 ± 0.08	0.70 ± 0.08	**0.013**
L-isoleucine degradation	0.05 ± 0.04	0.10 ± 0.03	**0.013**
L-methionine biosynthesis	0.11 ± 0.05	0.17 ± 0.05	**0.046**
Piruvate fermentation to acetate	0.01 ± 0.01	0.02 ± 0.02	**0.034**
Succinate fermentation to butanoate	0.08 ± 0.03	0.17 ± 0.03	**<0.001**
Superpathway of glycolysis, pyruvate dehydrogenase, TCA, and glyoxylate bypass	1.15 ± 0.20	0.84 ± 0.12	**<0.001**
Superpathway of L-homoserine and L-methionine biosynthesis	0.18 ± 0.06	0.25 ± 0.06	**0.044**
Superpathway of L-lysine, L-threonine and L-methionine biosynthesis II	0.03 ± 0.02	0.05 ± 0.02	**0.023**

* Calculated with a t test. Data expressed as the average percentage of the representation of the pathway on the overall metagenome of the samples of the same group. Significant *p* values (<0.05) are indicated in bold.

## Supplementary Materials

The following are available online at https://www.mdpi.com/1422-0067/21/23/8946/s1. Supplementary File S1—Full list of the bacterial species, with complete taxonomic classification, whose relative abundance was compared between sarcopenic and non-sarcopenic subjects who participated in the study. These species were selected as those having a relative abundance ≥0.1% in at least one fecal sample. Supplementary File S2—Full list of the MetaCyc pathways of the fecal metagenome whose expression was assessed in the fecal samples of study participants by shotgun metagenomics sequencing. The average representation and its standard deviation are indicated for sarcopenic subjects (SB) and non-sarcopenic controls (SBCT), together with p-values of comparisons obtained by the *t* test. The pathways with significantly different representation in SB and SBCT are listed first.

## Data Accessibility

Raw sequencing data have been deposited, in rigorously anonymous form, in the NCBI depository under the following ID: SUB8323288.

## Abbreviations

BMI	Body Mass Index
IQR	Interquartile Range
PCoA	Principal Coordinate Analysis
PF&S	Physical Frailty and Sarcopenia
SMI	Skeletal Muscle Index
SMM	Skeletal Muscle Mass
SPPB	Short Physical Performance Battery
